# Evolution and Spread of Highly Pathogenic Avian Influenza A(H5N1) Clade 2.3.4.4b Virus in Wild Birds, South Korea, 2022–2023

**DOI:** 10.3201/eid3002.231274

**Published:** 2024-02

**Authors:** Ye-Ram Seo, Andrew Y. Cho, Young-Jae Si, Song-I Lee, Dong-Ju Kim, Hyesung Jeong, Jung-Hoon Kwon, Chang-Seon Song, Dong-Hun Lee

**Affiliations:** Konkuk University, Seoul, South Korea (Y.-R. Seo, A.Y. Cho, C.-S. Song, D.-H. Lee);; National Institute of Wildlife Disease Control and Prevention, Gwangju, South Korea (Y.-J. Si, S.-I Lee, D.-J. Kim, Hyesung Jeong);; Kyungpook National University College of Veterinary Medicine, Daegu, South Korea (J.-H. Kwon)

**Keywords:** influenza, highly pathogenic avian influenza virus, H5N1, clade 2.3.4.4b, wild bird, South Korea, phylogenetic analysis, genomic surveillance, zoonoses

## Abstract

During October 2022–March 2023, highly pathogenic avian influenza (HPAI) A(H5N1) clade 2.3.4.4b virus caused outbreaks in South Korea, including 174 cases in wild birds. To understand the origin and role of wild birds in the evolution and spread of HPAI viruses, we sequenced 113 HPAI isolates from wild birds and performed phylogenetic analysis. We identified 16 different genotypes, indicating extensive genetic reassortment with viruses in wild birds. Phylodynamic analysis showed that the viruses were most likely introduced to the southern Gyeonggi-do/northern Chungcheongnam-do area through whooper swans (*Cygnus cygnus*) and spread southward. Cross-species transmission occurred between various wild bird species, including waterfowl and raptors, resulting in the persistence of HPAI in wild bird populations and further geographic spread as these birds migrated throughout South Korea. Enhanced genomic surveillance was an integral part of the HPAI outbreak response, aiding in timely understanding of the origin, evolution, and spread of the virus.

Since the first detection highly pathogenic avian influenza (HPAI) virus A/goose/Guangdong/1/1996 (Gs/Gd) in China in 1996, the Gs/Gd lineage of HPAI H5Nx virus has spread globally, infecting various species and posing a threat to animal and human health (*1*). Outbreaks caused by 1 Gs/Gd strain, clade 2.3.4.4, have occurred worldwide, and the clade has further evolved into 8 subclades (2.3.4.4a–2.3.4.4h) (*2*). Since 2013, ancestral HPAI H5 viruses of clade 2.3.4.4, in combination with different neuraminidase subtypes (e.g., H5N1, H5N6, and H5N8), have been circulating in Southeast Asia. Clade 2.3.4.4b H5N8 viruses were first detected in China in late 2013 and in South Korea in early 2014 (*3*). H5N8 viruses were detected in wild birds in Qinghai Lake, China, and Lake Uvs-Nuur, Russia, in May 2016 (*4*), and were then disseminated to Europe by wild birds (*5*). In 2020, a new variant of the clade 2.3.4.4b H5N1 virus emerged in Europe and spread predominantly through wild birds to various regions including Europe, Asia, and Africa (*6*–*8*). In late 2021, that 2.3.4.4b H5N1 virus spread to North America and subsequently to South America in 2022 (*9*). As of September 2023, H5N1 clade 2.3.4.4b virus is widespread in all regions except Antarctica and Oceania, causing infections and deaths in various wild birds and mammals (*10*) and posing major threats to public health and wildlife conservation.

In South Korea, 10 outbreaks of Gs/Gd-lineage H5Nx HPAI viruses have been recorded to date: clade 2.5 H5N1 outbreak during 2003–2004, clade 2.2 H5N1 during 2006–2007, clade 2.3.2 H5N1 in 2008, clade 2.3.2.1 H5N1 during 2010–2011, clade 2.3.4.4c H5N8 during 2014–2016, clade 2.3.4.4e H5N6 and clade 2.3.4.4c H5N8 during 2016–2017, clade 2.3.4.4b H5N6 during 2017–2018, clade 2.3.4.4b H5N8 during 2020–2021, clade 2.3.4.4b H5N1 during 2021–2022, and clade 2.3.4.4b H5N1 during 2022–2023 (*11*–*15*). Of those, the most recent H5N1 2.3.4.4b HPAI virus was first detected in a wild Mandarin duck in October 2022, and multiple outbreaks occurred in wild birds and poultry before their absence of detection after March 2023. During October 2022–March 2023, a total of 75 cases of HPAI infection in poultry farms and 174 cases of HPAI infection in wild birds were reported (*16*). The higher number of HPAI viruses detected in wild birds than in poultry probably reflected the higher level of virus circulation in wild birds, highlighting the need for further investigating the process underlying virus emergence and spread in wild birds. Therefore, we sequenced and analyzed 113 H5N1 HPAI virus isolates, which were collected by the Korean National Institute of Wildlife Disease Control and Prevention (NIWDC) under the national wild bird surveillance program in South Korea during 2022–2023, to identify the origin and reconstruct the evolutionary and transmission dynamics of H5N1 HPAI viruses in South Korea.

## Materials and Methods

### Samples and Spatial Distribution

The national wild bird surveillance program in South Korea has reported 174 HPAI H5N1 viruses during 2022–2023. We isolated HPAI H5N1 viruses from wild bird habitats and major streams during September 2022–February 2023 in South Korea during the Avian Influenza National Surveillance for the Protection and Management of Wildlife Animals and Plants, conducted by the Ministry of Environment. For active surveillance, we collected oropharyngeal, or cloacal swabs from captured wild birds and fecal samples from wild bird habitats. We tested carcasses of wild birds submitted voluntarily to NIWDC for HPAI. We transported, checked, and inoculated all samples in the allantoic cavities of 9–11-day-old specific-pathogen–free embryonated eggs at 37°C for 96 hours. We performed avian host identification for avian influenza–positive fecal samples by using DNA barcoding as previously described (*17*). For this study, we selected 113 HPAI H5N1 isolates for phylogenetic and phylodynamic analyses on the basis of their geographic locations and collection dates (Appendix Table 1). We analyzed spatial distribution of 113 HPAI H5N1 isolates by using the kernel model in ArcGIS software (ESRI, https://www.esri.com) to identify areas with a high density (Appendix Table 1).

### Genome Sequencing and Phylogenetic Analysis

We conducted next-generation sequencing (NGS) as previously described (*18*), and we deposited consensus genome sequences into the GISAID database (isolate nos. EPI_ISL 1824572–1824583 and EPI_ISL 18242686). To determine the genotypes, we constructed the maximum-likelihood phylogeny of each gene segment. We performed Bayesian phylodynamic analysis of geographic location and wild bird species. First, to investigate virus transmission between geographic locations, we defined geographic regions as 10 discrete nominal categories: Russia, Japan, China, and 7 locations in South Korea—Gyeonggi-do (GG), Gangwon-do (GW), Chungcheongbuk-do (CB), Chungcheongnam-do (CN), Gyeongsangbuk-do (GB), Gyeongsangnam-do (GN), and Jeolla-do (JL). Second, to infer the transmission dynamics between host species, host species were divided into 8 discrete nominal categories as follows: bean goose, crane, egret and heron, gull and crow, raptor, swan, white-fronted goose, and wild duck (Appendix).

## Results

### Overview of 2022–23 HPAI H5N1 Viruses from Wild Birds in South Korea

**Figure 1:**
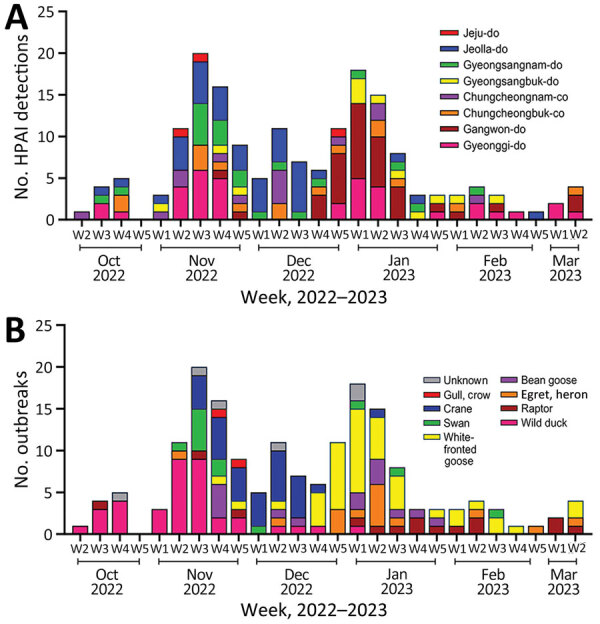
HPAI A(H5N1) clade 2.3.4.4b viruses and outbreaks, South Korea, October 2022–March 2023. A) Number of HPAI viruses detected in wild birds, by week, month, and geographic region. Geographic regions were determined according to a discrete trait analysis conducted for the study. B) Number of HPAI outbreaks, by week, month, and host species category. HPAI, highly pathogenic avian influenza.

In South Korea, the 2022–23 H5N1 HPAI outbreak started with virus detection in a wild Mandarin duck in October 2022 and lasted in wild birds until March 2023 (Figure 1). A total of 174 cases of infections in wild birds were reported in 132 carcasses, 31 fecal droppings, and 11 captured wild birds. The HPAI H5N1 virus was detected in 22 wild bird species, including 16 eastern spot-billed ducks (*Anas zonorhyncha*), 28 hooded cranes (*Grus monacha*), 44 greater white-fronted geese (*Anser albifrons*), 11 whooper swans (*Cygnus cygnus*), 10 Eastern great egrets (*Ardea modesta*), and 5 unspecified bird species.

Two major waves of outbreaks occurred, a peak in the third week of December, and the second highest number of outbreaks in the first week of January. The first wave of outbreaks mainly affected the GG and JL regions and involved species such as wild duck and crane. The second wave of outbreaks mainly affected the GW region and involved white-fronted goose species (Figure 1).

**Figure 2:**
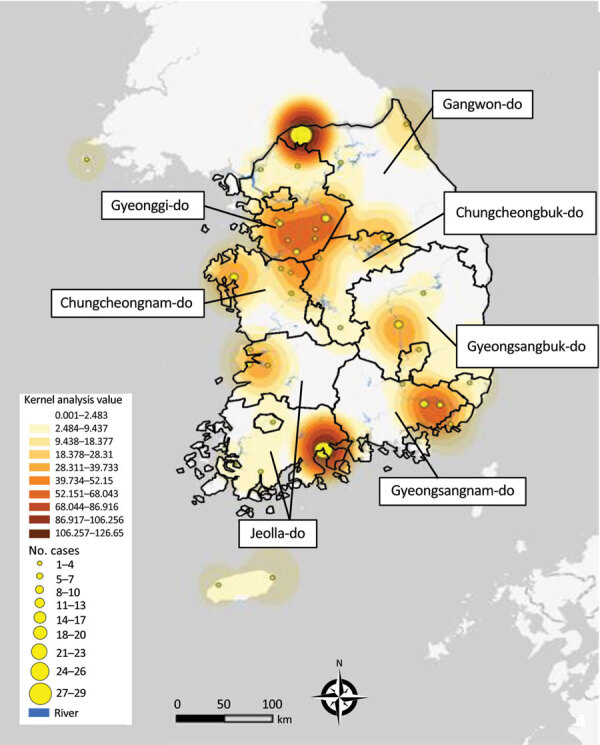
Kernel density map of epizootic cases of highly pathogenic avian influenza, by geographic region, South Korea, October 2022–March 2023. Geographic regions determined according to a discrete trait analysis conducted for the study.

We examined the spatial distribution of all HPAI outbreaks that occurred during 2022–2023 by using the kernel density estimation method. The results showed a clear geographic distribution of high-density areas around inland water bodies. We observed the peak distribution of kernel density values in GW and the highest concentration of cases in JL (Figure 2). Southern GG, northern CN, and northern CB, which are adjacent provinces that share inland water bodies, form a single high-density area.

### Origin of H5N1 HPAI Viruses

**Figure 3:**
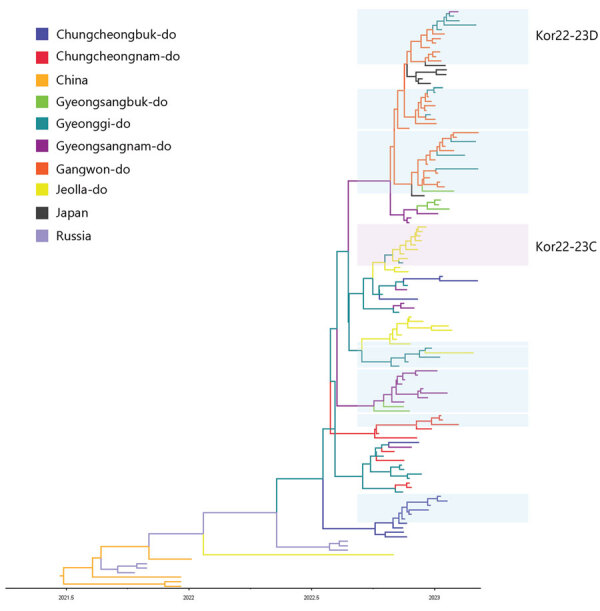
Maximum clade credibility tree constructed using the hemagglutinin gene of highly pathogenic avian influenza A(H5N1) clade 2.3.4.4b virus, with geographic region as a discrete trait, South Korea, June 2022–January 2023. Each branch is colored according to the geographic region specified in the legend. Each genotype was assigned an alphabet letter based on the Kor22–23 nomenclature, which indicated the region of origin (Kor) and year of origin (2022–2023). Orange shade represents Kor22–23B genotype viruses. Violet shades represent Kor22–23C genotype viruses. Blue shades represent Kor22–23D genotype viruses. The x-axis is in decimal year format.

The phylogenetic analysis of the hemagglutinin (HA) gene showed that all HPAI viruses sequenced in this study belonged to the 2.3.4.4b HPAI lineage. All of them carried a multi-basic amino acid motif at the HA cleavage site: PLREKRRKR/GLF (n = 106), PLRERRRKR/GLF (n = 2), PLRENRRKR/GLF (n = 2), PLREERRKRR/GLF (n = 1), PLREKRRRR/GLF (n = 1), and PLREKGRKR/GLF (n = 1). We hypothesized that the origin of clade 2.3.4.4b H5N1 viruses that caused outbreaks during 2022–23 was because of the reemergence of 2021–22 HPAI H5N1 viruses that persisted in wild bird populations in South Korea throughout the summer of 2022 or the introduction of the novel clade 2.3.4.4b H5N1 HPAI virus from outside South Korea. Our phylogenetic analysis results suggested that the latter assumption was the most likely one because the source-sink dynamics support that HPAI H5N1 virus was newly introduced into South Korea from Russia during the fall of 2022 (Figure 3; Appendix Table 2). The molecular dating analysis of the HA gene estimated the time to the most recent common ancestor of HPAI H5N1 viruses in South Korea to be July 14, 2022 (95% Bayesian credible interval [BCrI] May 11–September 5, 2022). The A/Em/Korea/22WF118–15P/2022 (H5N1) virus, which was detected on November 1, 2022, was not clustered with other H5N1 viruses identified in South Korea, suggesting a point-source introduction. The most closely related virus was A/Jiangsu/NJ210/2023 (H5N1), which infected humans in February 2023 in the Jiangsu region of China (Appendix Figure 1) (*19*).

### Genotypes

**Figure 4:**
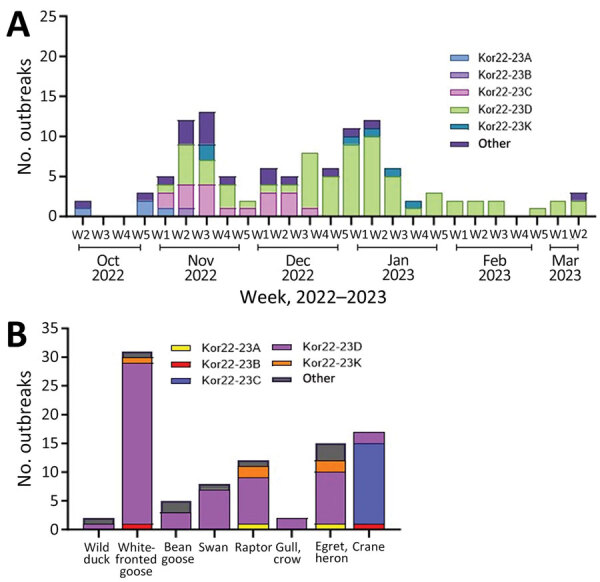
HPAI A(H5N1) clade 2.3.4.4b viruses and outbreaks, South Korea, October 2022–March 2023. A) Number of HPAI outbreaks by week, month, and genotype. B) Number of HPAI outbreaks, by week, month, and host species category. Each genotype was assigned an alphabet letter based on the Kor22–23 nomenclature, which indicated the region of origin (Kor) and year of origin (2022–2023). HPAI, highly pathogenic avian influenza.

The maximum-likelihood phylogenetic analysis of the 8 gene segments helped identify the genotypes of H5N1 viruses. We identified 16 unique genotypes, which were most likely produced by multiple reassortment events with low pathogenic avian influenza viruses in wild birds. We assigned each genotype an alphabet letter based on the Kor22–23 nomenclature, which indicated the region of origin (Kor) and year of origin (2022–2023). The 16 genotypes were as follows: Kor22–23A (n = 4), Kor22–23B (n = 2), Kor22–23C (n = 18), Kor22–23D (n = 65), Kor22–23E (n = 6), Kor22–23F (n = 1), Kor22–23G (n = 1), Kor22–23H (n = 1), Kor22–23I (n = 1), Kor22–23J (n = 2), Kor22–23K (n = 6), Kor22–23L (n = 1), Kor22–23M (n = 1), Kor22–23N (n = 1), Kor22–23O (n = 2), and Kor22–23P (n = 1) (Appendix Table 4). The predominant genotype was Kor22–23D, accounting for 57.5% of cases, followed by Kor22–23C, accounting 15% of cases. In those predominant genotypes (Kor22–23D and Kor22–23C), we observed reassortment in all internal genes, except for the matrix protein gene, compared with the early genotype, Kor22–23A. The most frequently identified internal genotypes were polymerase basic 2 (d), polymerase basic 1 (d), polymerase acidic (d), HA (a), nucleoprotein (d), neuraminidase (a), matrix protein (a), and nonstructural (d), which corresponded to the internal genes of the Kor22–23D genotype (Appendix Figure 1). Genotype diversity decreased from late December 2022, in particular as a consequence of the disappearance of Kor22–23C and Kor22–23B (Figure 4, panel A). Kor22–23D exhibited the highest proportions in GW, CN, and GN. Kor22–23C showed the highest proportion in JL. Kor22–23B was sporadically distributed across various regions.

### Transmission Dynamics of HPAI H5N1 Viruses in South Korea during 2022–23

**Figure 5:**
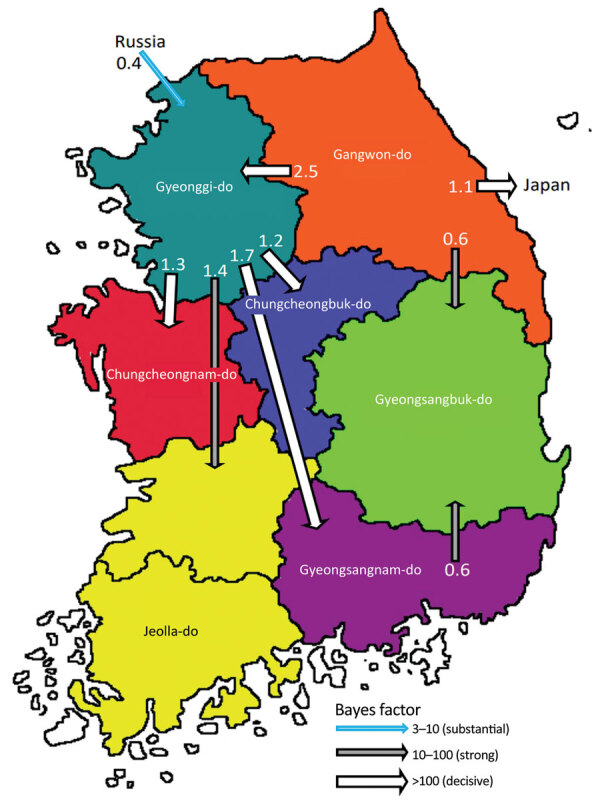
Inferred transmission networks of highly pathogenic avian influenza A(H5N1) viruses from wild birds, South Korea, October 2022–March 2023. Arrows show the well-supported transitions in discrete trait phylogeography. Line colors indicate the overall Bayes factor test support for epidemiologic linkage. White arrows indicate statistical support with Bayes factor > 100 (very strong support), gray arrows indicate support with 10 < Bayes factor < 100 (strong support), and cyan arrows indicate support with 3 < Bayes factor < 10. Numbers next to arrows indicate transition rates.

**Figure 6:**
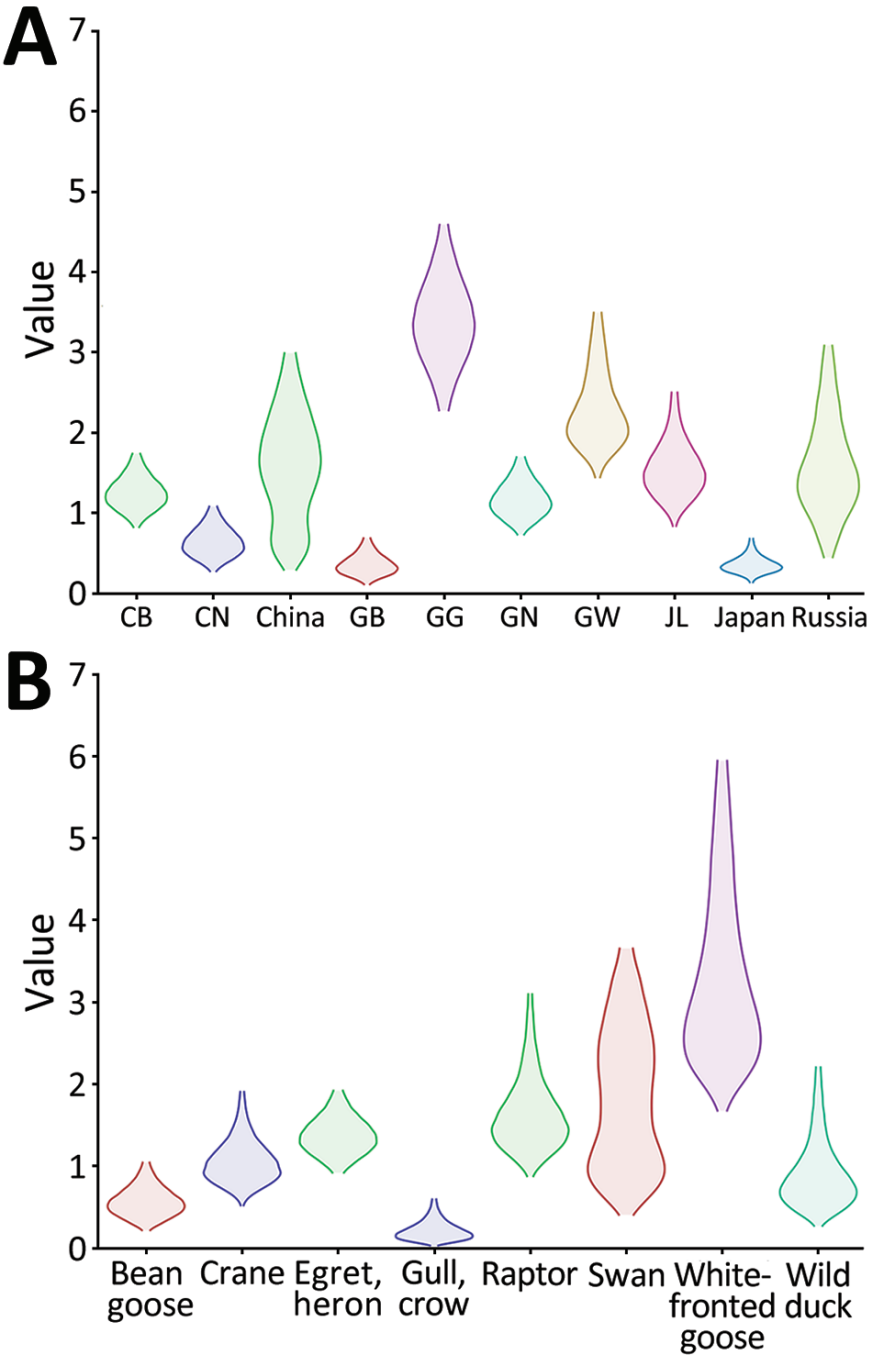
Markov time spent for geographic location (A) and host type (B) among highly pathogenic avian influenza A(H5N1) viruses from wild birds, South Korea, October 2022–March 2023. CB, Chungcheongbuk-do; CN, Chungcheongnam-do; GB, Gyeongsangbuk-do; GG, Gyeonggi-do; GN, Gyeongsangnam-do; GW, Gangwon-do; JL, Jeolla-do.

The discrete trait phylodynamic analyses (DTA) between geographic regions suggested that the virus was most likely dispersed from China to Russia during 2021–2022, then introduced from Russia to the GG of South Korea during 2022. In South Korea, the DTA supported the virus spread from GW to GG (transition rate [TR] 2.508, Bayes factor [BF] 212524.514, and posterior probability [PP] 1.00), GG to GN (TR 1.711, BF 35413.848, and PP 1.00), and GG to CN (TR 1.293, BF 284.057, and PP 0.97) (Appendix Table 2). We visualized the inferred transmission networks in South Korea on a map (Figure 5). We also inferred the time spent on each discrete state by estimating the Markov reward. The estimated total Markov reward time for locations in South Korea was highest in GG (3.407 [95% BCrI 2.237–4.6073]), followed by GW (2.371 [95% BCrI 1.4939–3.6048]), indicating the contribution of wild birds in GG and GW to virus persistence and circulation in South Korea (Figure 6, panel A).

**Figure 7:**
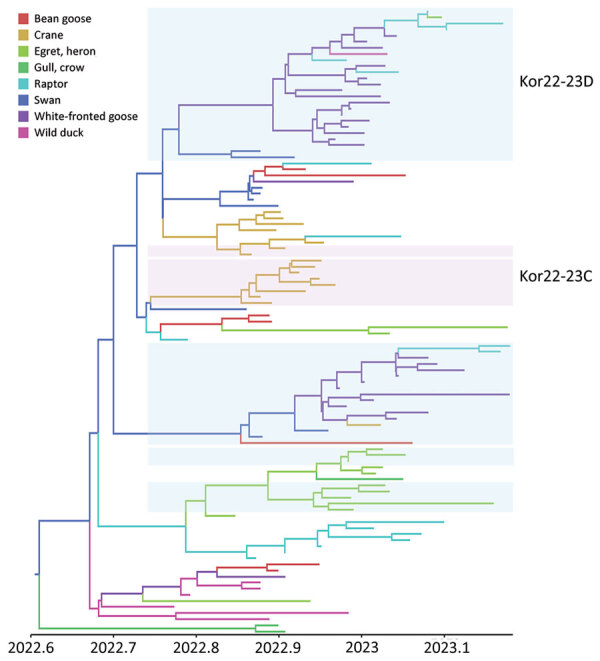
Maximum clade credibility tree constructed using the hemagglutinin gene of HPAI A(H5N1) clade 2.3.4.4b virus, with host types as a discrete trait, South Korea, June 2022–January 2023. Each branch is colored according to the host type specified in the legend. Each genotype was assigned an alphabet letter based on the Kor22–23 nomenclature, which indicated the region of origin (Kor) and year of origin (2022–2023). Orange shade represents Kor22–23B genotype viruses. Violet shades represent Kor22–23C genotype viruses. Blue shades represent Kor22–23D genotype viruses. The x-axis is in decimal year format.

On the basis of the DTA between wild bird species, the most probable transmission pathway was from white-fronted goose to raptor (TR 1.804, BF 710.953, and PP 0.99), followed by swan to bean goose (TR 1.041, BF 21.822, and PP 0.78) and swan to white-fronted goose (TR 1.233, BF 17.182, and PP 0.73) (Figure 7; Appendix Table 3). For genotypes, Kor22–23D was the predominant genotype, and its highest proportion was detected in white-fronted goose, egret, and heron, whereas the highest proportion of Kor22–23C was detected in crane. The raptor and bean goose contained highly diverse genotypes compared with those genotypes contained by other host species (Figure 4, panel B). Collectively, those results indicate that the white-fronted goose and swan played a major role in the HPAI H5N1 virus spread in South Korea. In addition, the estimated total Markov reward time by host species was the highest in white-fronted goose (3.428 [95% BCrI 1.7149–5.8995]) (Figure 6, panel B). Those findings suggest that the white-fronted goose contributed to virus maintenance and spread in South Korea during 2022–2023.

## Discussion

Understanding the movement and host characteristics of migratory waterfowl, which are natural hosts of avian influenza, is crucial for understanding the evolution and transmission pathways of HPAI virus. Migratory birds play a critical role in virus transmission because they have vast ranges, undertake long-distance migrations across borders to and from wintering and breeding sites, and interact with different species at each site (*20*). Some bird species can carry the virus without showing clinical signs of disease, which makes them long-distance carriers of the virus (*21*). Moreover, migratory waterfowl can potentially bring diverse strains of viruses, facilitating the emergence of new reassortant strains. Clade 2.3.4.4 HPAI viruses have undergone frequent reassortment events, especially in wild birds (*7*). The high genetic diversity of avian influenza viruses in wild birds has contributed to the generation of multiple genotypes of clade 2.3.4.4. HPAI H5Nx viruses as a donor gene pool of different genetic lineages (*22*). In our study, the genotyping of the 113 wild bird–origin HPAI H5N1 clade 2.3.4.4b viruses observed during October 2022–March 2023 revealed 16 different genotypes. Most of these newly reassorted genotypes were transient and did not show sustained transmission in wild bird populations, except for Kor22–23D and Kor22–23C. We assume that those 2 genotypes were predominant during the outbreak because they had better viral fitness than other transient genotypes for sustained transmission in wild birds.

The DTA between geographic locations indicated that most probable location for initial virus introduction to South Korea was GG. DTA is used in molecular epidemiology to study the geographic spread of infectious diseases across regions. When applied at the provincial or state level, a major limitation of this approach is its reliance on manmade borders as a proxy for the geographic structure. Manmade borders, such as provinces or state lines, do not necessarily align with the natural distribution of wild bird populations or movement patterns of viruses. Using such borders may lead to oversimplification and inaccuracies in analyses because they may not reflect the actual transmission routes or wild bird movements. Our DTA results suggest that the virus was introduced from Russia to GG; however, the actual index case in South Korea was identified in the northern part of the CN. The kernel density analysis showed that southern GG and northern CN were classified as single high HPAI-density areas. Based on these findings, the virus was most likely introduced into the southern GG and northern CN areas initially and then spread to other regions. In addition, an HPAI H5N1 virus originating from the North American lineage was detected in October 2022, which is most likely a point-source introduction of virus (*23*).

Monitoring the geographic distribution of HPAI viruses in wild birds and locations of poultry farms is vital for assessing the risk for virus spillover. A previous study analyzed the geographic distribution of HPAI H5N1 outbreaks in poultry farms in South Korea during 2013–2017 and found high number of cases in southern GG, northern CN, and JL (*24*), which were also high-density areas of HPAI detections in wild birds during 2022–2023. In particular, JL is the region with the highest poultry farm density in South Korea (*25*). JL is also the region with the highest number of HPAI H5N1 outbreaks in poultry farms during winter 2022–2023, according to the South Korean Animal and Plant Quarantine Agency (*16*). In addition, most HPAI H5N8 transmissions occurred during 2014–2015 within the western provinces, including GG, CN, and JL, which are characterized by high domestic duck densities and high numbers of overwintering waterfowl (*26*). On the basis of those findings, we suggest that integrative analysis of real-time surveillance data in wild birds and spatial distribution data of poultry farms can serve as an early warning system for forecasting the risk for avian influenza spillover from wild birds to poultry.

A previous study suggested the dissemination of HPAI viruses between South Korea and Japan through wild birds (*11*); however, the means by which the virus spreads between these 2 countries remains unclear. Of note, our DTA suggests that, after the virus spread to GW, it migrated from GW to central Japan, including Yamagata, Tochigi, Miyagi, and Shizuoka Prefectures, which have similar latitudes to that of GW. Further studies on the virus spread through migratory birds between GW and central Japan may provide valuable insights into the transboundary spread of HPAI viruses between these countries.

The initial entry of the virus through whooper swans was highly supported by DTA of host species. Whooper swans have been previously reported as long-distance migratory birds that can transmit HPAI viruses along their migration route. Previous studies on satellite GPS data showed that whooper swans bred in Mongolia and Russia were positive for HPAI viruses and subsequently migrated to South Korea, which coincides with the viral movement observed in our study (*27*). Considering the limitations of sampling in our surveillance study, an alternative explanation for this finding could be that the high susceptibility and distinctive morphology of whooper swans facilitated their early detection and subsequent sampling, potentially leading to the identification of HPAI viruses during the initial stages of the outbreak. Given that the most samples used in our surveillance study were wild bird carcasses collected by NIWDC through voluntary reports, asymptomatic wild bird carriers of HPAI viruses might not have been detected or included in our study. Of note, among the wild bird species affected by HPAI H5 virus, whooper swans have been reported as a frequently affected species thus recognized as a sentinel species for the presence of HPAI virus within the wild bird population (*27*). The frequent detections of swans found dead from HPAI infection can be attributed to their pathobiologic and morphologic characteristics compared with other wild bird species, including their high susceptibility to HPAI H5 virus infection and their substantial size and white color, which make them readily noticeable and identifiable when they are found dead from HPAI infection (*28*). Although passive surveillance of wild birds is considered the most effective method for monitoring HPAI associated with high mortality rates, active surveillance should be expanded in asymptomatic host species to improve our understanding of the behavior of these viruses in the wild-bird population. The DTA results suggested that geese (bean goose and white-fronted goose) played a crucial role in the virus spread in South Korea after these viruses were introduced by swans. Transmission was relatively frequent among swans, geese, and cranes, likely because those species share habitats around inland water bodies in South Korea (*29*). In addition, transmission of viruses from geese to raptors suggests possible upward food chain transmission during the predation of possibly infected geese.

NGS is a useful tool for obtaining complete genome sequences that can be used for outbreak analysis, combined with epidemiologic investigation data, to identify the possible origins and understand the transmission dynamics of viruses rapidly and accurately. During the 2022–23 HPAI virus outbreak, NIWDC attempted to sequence all available HPAI virus isolates from wild birds by using NGS and performed a comparative phylogenetic analysis to facilitate the rapid screening of HPAI and effective genomic surveillance. The 113 genome sequences identified in our study greatly expanded the existing dataset of the genome sequences of clade 2.3.4.4b HPAI virus from South Korea and helped us investigate the evolutionary history and molecular epidemiology of the virus outbreak across spatial and temporal scales. Immediate genome sequencing and analysis during outbreaks are recommended as an integral part of the HPAI virus outbreak response because this information can provide valuable insights into the origin, evolution, and spread of the virus. The precise and detailed genomic surveillance data can be especially beneficial to outbreak control and prevention efforts by helping public health officials and researchers monitor the emergence of new variants, trace the transmission of HPAI virus, tailor interventions and recommendations for the public, and develop countermeasures. Sharing genomic data with the global scientific community and international health organizations can foster collaboration and a coordinated response to global HPAI outbreaks.

Appendix Additional information about evolution and spread of highly pathogenic avian influenza A(H5N1) clade 2.3.4.4b virus in wild birds, South Korea, 2022–2023.

## References

[1] Wan XF. Lessons from emergence of A/goose/Guangdong/1996-like H5N1 highly pathogenic avian influenza viruses and recent influenza surveillance efforts in southern China. Zoonoses Public Health. 2012;59(Suppl 2):32–42. 10.1111/j.1863-2378.2012.01497.x 22958248 PMC4119829

[2] World Health Organization. Antigenic and genetic characteristics of zoonotic influenza A viruses and development of candidate vaccine viruses for pandemic preparedness. Wkly Epidemiol Rec. 2020;95:525–39.

[3] Lee YJ, Kang HM, Lee EK, Song BM, Jeong J, Kwon YK, et al. Novel reassortant influenza A(H5N8) viruses, South Korea, 2014. Emerg Infect Dis. 2014;20:1087–9. 10.3201/eid2006.140233 24856098 PMC4036756

[4] Lee DH, Sharshov K, Swayne DE, Kurskaya O, Sobolev I, Kabilov M, et al. Novel reassortant clade 2.3.4.4 avian influenza A(H5N8) virus in wild aquatic birds, Russia, 2016. Emerg Infect Dis. 2017;23:359–60. 10.3201/eid2302.161252 27875109 PMC5324796

[5] Beerens N, Heutink R, Bergervoet SA, Harders F, Bossers A, Koch G. Multiple reassorted viruses as cause of highly pathogenic avian influenza A(H5N8) virus epidemic, the Netherlands, 2016. Emerg Infect Dis. 2017;23:1974–81. 10.3201/eid2312.171062 29148396 PMC5708218

[6] Bevins SN, Shriner SA, Cumbee JC Jr, Dilione KE, Douglass KE, Ellis JW, et al. Intercontinental movement of highly pathogenic avian influenza A(H5N1) clade 2.3.4.4 virus to the United States, 2021. Emerg Infect Dis. 2022;28:1006–11. 10.3201/eid2805.220318 35302933 PMC9045435

[7] Baek YG, Lee YN, Lee DH, Shin JI, Lee JH, Chung DH, et al. Multiple reassortants of H5N8 clade 2.3.4.4b highly pathogenic avian influenza viruses detected in South Korea during the winter of 2020–2021. Viruses. 2021;13:490. 10.3390/v13030490 33809549 PMC8001867

[8] Wu H, Peng X, Xu L, Jin C, Cheng L, Lu X, et al. Novel reassortant influenza A(H5N8) viruses in domestic ducks, eastern China. Emerg Infect Dis. 2014;20:1315–8. 10.3201/eid2008.140339 25075453 PMC4111196

[9] Ruiz-Saenz J, Martinez-Gutierrez M, Pujol FH. Multiple introductions of highly pathogenic avian influenza H5N1 clade 2.3.4.4b into South America. Travel Med Infect Dis. 2023;53:102591. 10.1016/j.tmaid.2023.102591 37201592

[10] Wille M, Klaassen M. No evidence for HPAI H5N1 2.3.4.4b incursion into Australia in 2022. Influenza Other Respir Viruses. 2023;17:e13118. 10.1111/irv.13118 36909297 PMC9995809

[11] Jeong S, Lee DH, Kwon JH, Kim YJ, Lee SH, Cho AY, et al. Highly pathogenic avian influenza clade 2.3.4.4b subtype H5N8 virus isolated from Mandarin duck in South Korea, 2020. Viruses. 2020;12:1389. 10.3390/v12121389 33291548 PMC7761861

[12] Shin J, Kang S, Byeon H, Cho SM, Kim SY, Chung YJ, et al. Highly pathogenic H5N6 avian influenza virus subtype clade 2.3.4.4 indigenous in South Korea. Sci Rep. 2020;10:7241. 10.1038/s41598-020-64125-x 32350323 PMC7190616

[13] Sagong M, Lee YN, Song S, Cha RM, Lee EK, Kang YM, et al. Emergence of clade 2.3.4.4b novel reassortant H5N1 high pathogenicity avian influenza virus in South Korea during late 2021. Transbound Emerg Dis. 2022;69:e3255–60. 10.1111/tbed.14551 35413157

[14] Baek YG, Lee YN, Park YR, Chung DH, Kwon JH, Si YJ, et al. Evolution, transmission, and pathogenicity of high pathogenicity avian influenza virus A (H5N8) clade 2.3.4.4, South Korea, 2014–2016. Front Vet Sci. 2022;9:906944. 10.3389/fvets.2022.906944 35799844 PMC9253604

[15] Song BM, Lee EK, Lee YN, Heo GB, Lee HS, Lee YJ. Phylogeographical characterization of H5N8 viruses isolated from poultry and wild birds during 2014-2016 in South Korea. J Vet Sci. 2017;18:89–94. 10.4142/jvs.2017.18.1.89 28316230 PMC5366307

[16] Animal and Plant Quarantine Agency (South Korea). Highly pathogenic avian influenza detection status. 2023 [cited 2023 Nov 5]. https://www.qia.go.kr/viewwebQiaCom.do?id=60620&type=2_12qlgzls

[17] Lee DH, Lee HJ, Lee YJ, Kang HM, Jeong OM, Kim MC, et al. DNA barcoding techniques for avian influenza virus surveillance in migratory bird habitats. J Wildl Dis. 2010;46:649–54. 10.7589/0090-3558-46.2.649 20688667

[18] Lee DH. Complete genome sequencing of influenza A viruses using next-generation sequencing. Methods Mol Biol. 2020;2123:69–79. 10.1007/978-1-0716-0346-8_6 32170681

[19] World Health Organization. Genetic and antigenic characteristics of zoonotic influenza A viruses and development of candidate vaccine viruses for pandemic preparedness. Wkly Epidemiol Rec. 2023;98:111–25.

[20] Olsen B, Munster VJ, Wallensten A, Waldenström J, Osterhaus AD, Fouchier RA. Global patterns of influenza a virus in wild birds. Science. 2006;312:384–8. 10.1126/science.1122438 16627734

[21] Tian H, Zhou S, Dong L, Van Boeckel TP, Cui Y, Newman SH, et al. Avian influenza H5N1 viral and bird migration networks in Asia. Proc Natl Acad Sci U S A. 2015;112:172–7. 10.1073/pnas.1405216112 25535385 PMC4291667

[22] Claes F, Morzaria SP, Donis RO. Emergence and dissemination of clade 2.3.4.4 H5Nx influenza viruses-how is the Asian HPAI H5 lineage maintained. Curr Opin Virol. 2016;16:158–63. 10.1016/j.coviro.2016.02.005 26991931

[23] Kang Y-M, Heo G-B, An S-H, Lee Y-N, Cha RM, Cho H-K, et al. Introduction of multiple novel high pathogenicity avian influenza (H5N1) virus of clade 2.3.4.4 b into South Korea in 2022. Transbound Emerg Dis. 2023;2023. 10.1111/tbed.14551 35413157

[24] Yoo DS, Chun BC, Hong K, Kim J. Risk prediction of three different subtypes of highly pathogenic avian influenza outbreaks in poultry farms: based on spatial characteristics of infected premises in South Korea. Front Vet Sci. 2022;9:897763. 10.3389/fvets.2022.897763 35711796 PMC9194674

[25] Koh K-Y, Ahmad S, Lee J-i, Suh G-H, Lee C-M. Hierarchical clustering on principal components analysis to detect clusters of highly pathogenic avian influenza subtype H5N6 epidemic across South Korean Poultry Farms. Symmetry (Basel). 2022;14:598. 10.3390/sym14030598

[26] Hill SC, Lee YJ, Song BM, Kang HM, Lee EK, Hanna A, et al. Wild waterfowl migration and domestic duck density shape the epidemiology of highly pathogenic H5N8 influenza in the Republic of Korea. Infect Genet Evol. 2015;34:267–77. 10.1016/j.meegid.2015.06.014 26079277 PMC4539883

[27] Li S, Meng W, Liu D, Yang Q, Chen L, Dai Q, et al. Migratory whooper swans *Cygnus cygnus* transmit H5N1 virus between China and Mongolia: combination evidence from satellite tracking and phylogenetics analysis. Sci Rep. 2018;8:7049. 10.1038/s41598-018-25291-1 29728621 PMC5935751

[28] Li X, Lv X, Li Y, Xie L, Peng P, An Q, et al. Emergence, prevalence, and evolution of H5N8 avian influenza viruses in central China, 2020. Emerg Microbes Infect. 2022;11:73–82. 10.1080/22221751.2021.2011622 34825854 PMC8725850

[29] Moores N. Wetlands: Korea’s most threatened habitat. Oriental Bird Club Bulletin. 2002;36:54–60.

