# Supplementary material for: Evolution and Spread of Highly Pathogenic Avian Influenza A(H5N1) Clade 2.3.4.4b Virus in Wild Birds, South Korea, 2022–2023
Source: Emerg Infect Dis. 2024 Feb;30(2):299–309. doi: 10.3201/eid3002.231274 (PMC10826760; doi:10.3201/eid3002.231274)
Supplement: Appendix — Additional information about evolution and spread of highly pathogenic avian influenza A(H5N1) clade 2.3.4.4b virus in wild birds, South Korea, 2022–2023. [file 23-1274-Techapp-s1.pdf]

Article DOI: https://doi.org/10.3201/eid3002.231274

EID cannot ensure accessibility for supplementary materials supplied by authors. Readers who have difficulty accessing supplementary content should contact the authors for assistance.

Evolution and Spread of Highly Pathogenic Avian Influenza A (H5N1) Clade 2.3.4.4b Virus in Wild Birds, South Korea, 2022–2023

Appendix

Methods

Genome Sequencing

The viral RNAs of isolates were extracted from allantoic fluids of SPF chicken eggs using Maxwell® RSC simply RNA Tissue Kit (Promega, Madison, WI, USA). Complementary DNA was generated using the SuperScript III First-Strand Synthesis system (Invitrogen, Carlsbad, CA, USA). Polymerase chain reaction was performed using AccuPrime Pfx DNA Polymerase (Invitrogen, Carlsbad, CA, USA) per the protocol (*1*). DNA libraries were prepared using Nextera DNA Flex Library Prep Kit (Illumina, San Diego, CA, USA) with 96 dual-index barcodes according to the manufacturer's instructions. The complete genome was sequenced with a minimum sequence coverage of 1,000x using the paired-end 150 Illumina Miseq platform. NGS data obtained were analyzed using CLC Genomic Workbench (Qiagen, Valencia, CA, USA). The consensus genome sequences have been deposited in the GISAID database (isolate ID: EPI_ISL 1824572 – 1824583 and EPI_ISL 18242686).

Phylogenetic analysis

A total of 2158 reference sequences for maximum likelihood (ML) phylogenetic analysis were obtained from the Global Initiative on Sharing All Influenza Data (GISAID, https://www.gisaid.org/) database. All available complete genome sequences of H5N1 2.3.4.4b identified since 2018 were downloaded from the GISAID database and redundant sequences were removed at the 100% nucleotide sequence identity level using the CD-HIT program (*2*). To

determine the genotypes, the ML phylogeny of each gene segment (PB2, PB1, PA, HA, NP, NA, M, and NS) was constructed using RAxML v8.0 (https://cme.h-its.org/exelixis/web/software/raxml/) to determine the best tree via 1,000 bootstrap analyses using the general time-reversible + Gamma model. The resulting phylogenetic trees were visualized using the online tool iTOL v5 (https://itol.embl.de/) (*3*). Monophyletic clades with a bootstrap support of 70% or higher were considered well-supported (*4*).

Phylodynamic analysis of geographic location and host type

We generated an ML phylogeny of the HA gene using FastTree version 2.1.11 (http://www.microbesonline.org/fasttree/). Root-to-tip regression was analyzed to investigate the temporal signal in the ML phylogeny using TempEst version 1.5.3 (http://tree.bio.ed.ac.uk/software/tempest/). From the completed FastTree Phylogeny, we extracted clades that contained the NIWDC data and organized two datasets to analyze transmission between geographic locations and between host species. Genome sequences of viruses originating from wild birds in countries that share the East Asian–Australasian Flyway were included in the analysis. Sequences were subsampled to preserve the diversity of geographic locations and host species, resulting in 129 and 103 sequences, respectively. We performed two separate Bayesian discrete trait phylodynamic analyses using BEAST version 1.10.4 (https://beast.community). First, to investigate virus transmission between geographic locations, we defined geographic regions as 10 discrete nominal categories as follows: “Russia” (n = 6), “Japan” (n = 6), “China” (n = 4), “GG” (Gyeonggi-do: proximal region encompassing Seoul, n = 24), “GW” (Gangwon-do: Northeast province of South Korea, n = 24), “CB” (Chungcheongbuk-do: landlocked central province of South Korea, n = 14), “CN” (Chungcheongnam-do: central Western province of South Korea, n = 10), “GB” (Gyeongsangbuk-do: Southeast province of South Korea, n = 6), “GN” (Gyeongsangnam-do: South Southeast province of South Korea, n = 16), and “JL” (Jeolla-do: region covering both Jeollabuk-do and Jeollanam-do, Southwestern region of South Korea, n = 19). Second, to infer the transmission dynamics between host species, host species were divided into eight discrete nominal categories as follows: “Bean Goose” (n = 7), “Crane” (n = 16), “Egret and Heron” (n = 15), “Gull and Crow” (n = 3), “Raptor” (n = 17), “Swan” (n = 9), “White-fronted Goose” (n = 29), and “Wild Duck” (n = 7).

For both analyses, the Hasegawa–Kishono–Yano (HKY) + Gamma asymmetric substitution model with an uncorrelated lognormal relaxed molecular clock was used in addition to a constant-size coalescent tree. An ancestral state reconstruction approach with a Bayesian stochastic search variable selection was used to determine the most probable transmission history. Bayesian Markov chain Monte Carlo (MCMC) was run in parallel for five chains with a chain length of 50 million. The chains were combined after a 10% burn-in to achieve reliable convergence and stationarity. The convergence and stationarity of MCMC chains were assessed using the effective sample size (ESS) in Tracer version 1.7.2 (http://tree.bio.ed.ac.uk/software/tracer/). Phylogenetic estimates were accepted if the ESSs of all estimates were higher than 200. Bayes factor (BF) was calculated using SPREAD v. 1.0.7 (*5*). BF >6 and posterior probability (PP) >0.5 were considered significant. A maximum clade credibility tree was generated using TreeAnnotator version 1.10.4 and visualized using FigTree version 1.4.4 (http://tree.bio.ed.ac.uk/software/figtree/). The transmission network was plotted on a chord diagram for better visualization.

References

1. Zhou B, Donnelly ME, Scholes DT, St George K, Hatta M, Kawaoka Y, et al. Single-reaction genomic amplification accelerates sequencing and vaccine production for classical and swine origin human influenza a viruses. J Virol. 2009;83:10309–13. PubMed https://doi.org/10.1128/JVI.01109-09

2. Fu L, Niu B, Zhu Z, Wu S, Li W. CD-HIT: accelerated for clustering the next-generation sequencing data. Bioinformatics. 2012;28:3150–2. PubMed https://doi.org/10.1093/bioinformatics/bts565

3. Letunic I, Bork P. Interactive Tree Of Life (iTOL) v5: an online tool for phylogenetic tree display and annotation. Nucleic Acids Res. 2021;49(W1):W293–6. PubMed https://doi.org/10.1093/nar/gkab301

4. Hillis DM, Bull JJ. An Empirical Test of Bootstrapping as a method for assessing confidence in phylogenetic analysis. Syst Biol. 1993;42:182–92. https://doi.org/10.1093/sysbio/42.2.182

5. Bielejec F, Rambaut A, Suchard MA, Lemey P. SPREAD: spatial phylogenetic reconstruction of evolutionary dynamics. Bioinformatics. 2011;27:2910–2. PubMed https://doi.org/10.1093/bioinformatics/btr481

Appendix Table 1. Information of clade 2.3.4.4b H5N1 high pathogenicity avian influenza virus isolated in this study

Number	Collection date	Sample ID	Region	Sample type	Host species
2	2022–10–16	22WC013	Gyeonggi-do (GG)	Carcass	Hawk
3	2022–10–17	22WS010–2	Gyeonggi-do (GG)	Captured	Eastern spot-billed duck
11	2022–11–01	22WF118–15P	Jeolla-do (JL)	Feces	Eastern spot-billed duck/Mallard
13	2022–11–02	22WF123–24P	Chungcheongnam-do (CN)	Feces	Eastern spot-billed duck/Mallard
14	2022–11–06	22WC022	Jeolla-do (JL)	Carcass	Eastern great egret
22	2022–11–09	22WF157–9P	Gyeonggi-do (GG)	Feces	Eastern spot-billed duck/Mallard
23	2022–11–11	22WC025	Jeolla-do (JL)	Carcass	Whooper swan
24	2022–11–09	22WF154–5P	Gyeonggi-do (GG)	Feces	-
25	2022–11–13	22WC026	Jeolla-do (JL)	Carcass	Hooded crane
29	2022–11–13	22WF167–12P	Gyeonggi-do (GG)	Feces	-
27	2022–11–14	22WC28–1P	Gyeongsangnam-do (GN)	Carcass	Whooper swan
30	2022–11–14	22WF171–1P	Jeju (JJ)	Feces	-
28	2022–11–15	22WC032	Chungcheongbuk-do (CB)	Carcass	Eurasian eagle owl
32	2022–11–15	22WF181–20P	Gyeonggi-do (GG)	Feces	-
33	2022–11–15	22WF182–9P	Gyeonggi-do (GG)	Feces	-
38	2022–11–17	22WC040	Gyeongsangnam-do (GN)	Carcass	Whooper swan
48	2022–11–21	22WC058	Gyeongsangnam-do (GN)	Carcass	Bean goose
37	2022–11–17	22WC037	Gyeongsangnam-do (GN)	Carcass	Whooper swan
39	2022–11–17	22WC042–1P	Jeolla-do (JL)	Carcass	Hooded crane
40	2022–11–18	22WC044	Chungcheongbuk-do (CB)	Carcass	Whooper swan
41	2022–11–18	22WC045	Gyeongsangnam-do (GN)	Carcass	Whooper swan
42	2022–11–18	22WC046–2	Jeolla-do (JL)	Carcass	Hooded crane
43	2022–11–16	22WF191–23P	Chungcheongbuk-do (CB)	Feces	-
49	2022–11–22	22WC064	Jeolla-do (JL)	Carcass	Hooded crane
50	2022–11–22	22WC068	Gyeonggi-do (GG)	Carcass	Bean goose
51	2022–11–21	22WF205–13P	Chungcheongbuk-do (CB)	Feces	-
52	2022–11–23	22WC071	Gyeonggi-do (GG)	Carcass	Common Cormorant
53	2022–11–24	22WC073	Gyeongsangnam-do (GN)	Carcass	Hooded crane
54	2022–11–23	22WF229–3P	Jeolla-do (JL)	Feces	-
57	2022–11–25	22WC077	Chungcheongnam-do (CN)	Carcass	Black-headed gull
60	2022–11–24	22WC099	Gangwon-do (GW)	Carcass	White-fronted goose
56	2022–11–25	22WC079	Gyeonggi-do (GG)	Carcass	Bean goose
58	2022–11–25	22WC086	Gyeongsangnabuk-do (GB)	Carcass	Swan
59	2022–11–26	22WC083	Jeolla-do (JL)	Carcass	Hooded crane
61	2022–11–27	22WC091	Gyeongsangnam-do (GN)	Carcass	White-naped crane
63	2022–11–28	22WC116	Gyeongsangnam-do (GN)	Carcass	White-fronted goose
64	2022–11–28	22WC105	Chungcheongbuk-do (CB)	Carcass	Eurasian eagle owl
65	2022–11–28	22WC109	Jeolla-do (JL)	Carcass	Hooded crane
66	2022–11–28	22WC117	Chungcheongnam-do (CN)	Carcass	Black-headed gull
68	2022–11–30	22WF252–7P	Gyeongsangnabuk-do (GB)	Feces	-
71	2022–12–02	22WC146	Gyeongsangnam-do (GN)	Carcass	Whooper swan
74	2022–12–04	22WC154	Jeolla-do (JL)	Carcass	Hooded crane
79	2022–12–06	22WC167	Chungcheongnam-do (CN)	Carcass	Hooded crane
81	2022–12–07	22WC177	Jeolla-do (JL)	Carcass	Hooded crane
82	2022–12–07	22WC178	Gyeongsangnam-do (GN)	Carcass	Bean goose
83	2022–12–07	22WF296–4P	Chungcheongbuk-do (CB)	Feces	-
84	2022–12–09	22WC188	Chungcheongbuk-do (CB)	Carcass	Eastern great egret
86	2022–12–11	22WC196	Jeolla-do (JL)	Carcass	Hooded crane
87	2022–12–13	22WC200	Gyeonggi-do (GG)	Carcass	Bean goose
88	2023–12–13	22WC-208	Jeolla-do (JL)	Carcass	Hooded crane
89	2023–12–14	22WC211	Jeolla-do (JL)	Carcass	Hooded crane
91	2023–12–15	22WC-215	Jeolla-do (JL)	Carcass	Hooded crane
92	2023–12–15	22WF-340–11p	Gyeongsangnam-do (GN)	Feces	-
93	2023–12–20	22WC-233	Gangwon-do (GW)	Carcass	White-fronted goose
94	2023–12–20	22WC-235	Jeolla-do (JL)	Carcass	Hooded crane
95	2023–12–22	22WF-378–1p	Gyeongsangnam-do (GN)	Feces	-
96	2023–12–23	22WC-244	Chungcheongbuk-do (CB)	Carcass	White-fronted goose
97	2023–12–23	22WC-250	Gangwon-do (GW)	Carcass	White-fronted goose
98	2023–12–22	22WC-254	Gangwon-do (GW)	Carcass	White-fronted goose
99	2023–12–25	22WC-247	Gangwon-do (GW)	Carcass	Buzzard
100	2023–12–25	22WC-252	Gangwon-do (GW)	Carcass	White-fronted goose
101	2023–12–26	22WC-245	Gyeonggi-do (GG)	Carcass	White-fronted goose
102	2023–12–26	22WC-265	Chungcheongbuk-do (CB)	Carcass	Eastern great egret
103	2023–12–27	22WC-281	Jeju (JJ)	Carcass	Eastern great egret
104	2023–12–27	22WC-278	Gangwon-do (GW)	Carcass	White-fronted goose
105	2023–12–28	22WC-284	Gyeonggi-do (GG)	Carcass	Eastern great egret
106	2023–12–28	22WC-280	Chungcheongnam-do (CN)	Carcass	White-fronted goose
111	2023–01–03	22WC-317	Gangwon-do (GW)	Carcass	White-fronted goose

Number	Collection date	Sample ID	Region	Sample type	Host species
112	2023–01–02	22WC-326	Gangwon-do (GW)	Carcass	White-fronted goose
113	2023–01–02	22WC-328	Gangwon-do (GW)	Carcass	White-fronted goose
114	2023–01–02	22WC-329	Gangwon-do (GW)	Carcass	White-fronted goose
115	2023–01–02	22WF-429–2p	Gyeonggi-do (GG)	Feces	-
116	2023–01–03	22WC-324	Gangwon-do (GW)	Carcass	White-fronted goose
117	2023–01–04	22WC-345	Gangwon-do (GW)	Carcass	White-fronted goose
118	2023–01–05	22WC-336	Gyeongsangnam-do (GN)	Carcass	Buzzard
125	2023–01–07	22WC-357	Gyeongsangnabuk-do (GB)	Carcass	Little egret
126	2023–01–06	22WC-363	Gangwon-do (GW)	Carcass	White-fronted goose
127	2023–01–09	22WC-365	Gangwon-do (GW)	Carcass	White-fronted goose
128	2023–01–09	22WC-366	Gangwon-do (GW)	Carcass	White-naped crane
129	2023–01–10	22WC-372	Gyeongsangnabuk-do (GB)	Carcass	Little egret
130	2023–01–09	22WC-373	Gyeonggi-do (GG)	Carcass	White-fronted goose
131	2023–01–10	22WC-376	Chungcheongbuk-do (CB)	Carcass	Eastern great egret
132	2023–01–10	22WC-378	Gangwon-do (GW)	Carcass	White-fronted goose
133	2023–01–11	22WC-374	Chungcheongnam-do (CN)	Carcass	Grey heron
134	2023–01–12	22WC-390	Gyeonggi-do (GG)	Carcass	Eurasian teal
137	2023–01–13	22WC-401–3P	Gyeonggi-do (GG)	Carcass	White-fronted goose
139	2023–01–13	22WC-394	Chungcheongnam-do (CN)	Carcass	Eastern great egret
140	2023–01–11	22WC-405	Gangwon-do (GW)	Carcass	White-fronted goose
141	2023–01–13	22WC-406	Chungcheongbuk-do (CB)	Carcass	Eastern great egret
142	2023–01–16	22WC-413	Gangwon-do (GW)	Carcass	White-fronted goose
143	2023–01–16	22WC-412	Gangwon-do (GW)	Carcass	White-fronted goose
144	2023–01–17	22WC-421	Gangwon-do (GW)	Carcass	White-fronted goose
148	2023–01–20	22WC-426	Chungcheongbuk-do (CB)	Carcass	Grey heron
149	2023–01–20	22WC-427	Gyeongsangnam-do (GN)	Carcass	Bean goose
150	2023–01–22	22WC-428	Jeolla-do (JL)	Carcass	Goshawk
151	2023–01–23	22WC-432	Gyeongsangnabuk-do (GB)	Carcass	Bean goose
152	2023–01–27	22WC-453	Jeolla-do (JL)	Carcass	Common kestrel
153	2023–01–06	22WC-464	Gyeongsangnam-do (GN)	Carcass	Eagle
154	2023–01–30	22WC-460	Gyeongsangnabuk-do (GB)	Carcass	White-fronted goose
155	2023–01–30	22WC-468	Gyeonggi-do (GG)	Carcass	Buzzard
156	2023–01–30	22WC-471	Gangwon-do (GW)	Carcass	White-fronted goose
159	2023–02–03	22WC-491	Gangwon-do (GW)	Carcass	White-fronted goose
160	2023–02–05	22WC-492	Gyeongsangnam-do (GN)	Carcass	Grey heron
162	2023–02–06	22WC-502	Chungcheongnam-do (CN)	Carcass	Eurasian eagle owl
163	2023–02–07	22WC-500	Gyeonggi-do (GG)	Carcass	Eurasian eagle owl
164	2023–02–13	22WC-522	Gyeongsangnabuk-do (GB)	Carcass	White-fronted goose
165	2023–02–15	22WC-529	Gyeonggi-do (GG)	Carcass	White-fronted goose
168	2023–02–28	22WC-572	Jeolla-do (JL)	Carcass	Little egret
169	2023–03–03	22WC-586	Gyeonggi-do (GG)	Carcass	Long-eared owl
170	2023–03–04	22WC-590	Gyeonggi-do (GG)	Carcass	Eurasian eagle owl
171	2023–03–07	22WC-597	Gangwon-do (GW)	Carcass	Common kestrel
173	2023–03–07	22WC-599	Gyeonggi-do (GG)	Carcass	White-fronted goose
174	2023–03–06	22WC-603	Chungcheongbuk-do (CB)	Carcass	Eastern great egret

Appendix Table 2. Transmission matrix between geographic location

From	To	Mean actual migration rate	Bayes factor	Posterior probability
Gangwon-do (GW)	Gyeonggi-do (GG)	2.508	212524.514	1.00
Gyeonggi-do (GG)	Gyeongsangnam-do (GN)	1.711	35413.848	1.00
Gyeonggi-do (GG)	Chungcheongnam-do (CN)	1.293	284.057	0.97
Gangwon-do (GW)	Japan	1.096	197.061	0.96
Gyeonggi-do (GG)	Chungcheongbuk-do (CB)	1.171	103.105	0.93
Gyeonggi-do (GG)	Jeolla-do (JL)	1.392	70.606	0.89
China	Russia	0.789	23.859	0.74
Gangwon-do (GW)	Gyeongsangbuk-do (GB)	0.572	15.971	0.66
Gyeongsangnam-do (GN)	Gyeongsangbuk-do (GB)	0.59	11.273	0.58
Russia	Gyeonggi-do (GG)	0.445	8.197	0.50

Appendix Table 3. Transmission matrix between host types

From	To	Mean actual migration rate	Bayes factor	Posterior probability
White-fronted goose	Raptor	1.804	710.953	0.99
Swan	Bean goose	1.041	21.822	0.78
Swan	White-fronted goose	1.233	17.182	0.73
White-fronted goose	Crane	0.625	17.016	0.73
White-fronted goose	Wild duck	0.642	12.775	0.67
Raptor	Egret and Heron	0.628	7.138	0.53
Wild duck	Egret and Heron	0.623	6.593	0.51
Bean goose	Raptor	0.49	6.544	0.51

Appendix Table 4. Genotype of clade 2.3.4.4b H5N1 high pathogenicity avian influenza viruses isolated in this study

Number	Collection Date	Sample ID	Genotype	PB2	PB1	PA	HA	NP	NA	MP	NS
2	2022–10–16	22WC013	A	a	a	a	a	a	a	a	a
3	2022–10–17	22WS010–2	H	a	e	a	a	e	a	a	c
11	2022–11–01	22WF118–15P	A	a	a	a	a	a	a	a	a
13	2022–11–02	22WF123–24P	E	a	e	g	a	e	a	a	c
14	2022–11–06	22WC022	A	a	a	a	a	a	a	a	a
22	2022–11–09	22WF157–9P	C	c	c	c	a	c	a	a	c
23	2022–11–11	22WC025	D	d	d	d	a	d	a	a	d
24	2022–11–09	22WF154–5P	A	a	a	a	a	a	a	a	a
25	2022–11–13	22WC026	C	c	c	c	a	c	a	a	c
27	2022–11–14	22WC28–1P	D	d	d	d	a	d	a	a	d
28	2022–11–15	22WC032	F	a	d	a	a	f	a	a	e
29	2022–11–13	22WF167–12P	E	a	e	g	a	e	a	a	c
30	2022–11–14	22WF171–1P	C	c	c	c	a	c	a	a	c
32	2022–11–15	22WF181–20P	C	c	c	c	a	c	a	a	c
33	2022–11–15	22WF182–9P	G	a	d	d	a	d	a	a	d
37	2022–11–17	22WC037	D	d	d	d	a	d	a	a	d
38	2022–11–17	22WC040	D	d	d	d	a	d	a	a	d
39	2022–11–17	22WC042–1P	C	c	c	c	a	c	a	a	c
40	2022–11–18	22WC044	D	d	d	d	a	d	a	a	d
41	2022–11–18	22WC045	D	d	d	d	a	d	a	a	d
42	2022–11–18	22WC046–2	B	b	b	b	b	b	b	b	b
43	2022–11–16	22WF191–23P	I	e	d	g	a	g	a	c	e
48	2022–11–21	22WC058	J	c	a	c	a	c	a	a	a
49	2022–11–22	22WC064	C	c	c	c	a	c	a	a	c
50	2022–11–22	22WC068	J	c	a	c	a	c	a	a	a
51	2022–11–21	22WF205–13P	K	a	c	c	a	c	a	a	c
52	2022–11–23	22WC071	D	d	d	d	a	d	a	a	d
53	2022–11–24	22WC073	C	c	c	c	a	c	a	a	c
54	2022–11–23	22WF229–3P	C	c	c	c	a	c	a	a	c
56	2022–11–25	22WC079	E	a	e	g	a	e	a	a	c
57	2022–11–25	22WC077	D	d	d	d	a	d	a	a	d
58	2022–11–25	22WC086	L	e	b	e	a	g	a	a	a
59	2022–11–26	22WC083	C	c	c	c	a	c	a	a	c
60	2022–11–24	22WC099	K	a	c	c	a	c	a	a	c
61	2022–11–27	22WC091	D	d	d	d	a	d	a	a	d
63	2022–11–28	22WC116	E	a	e	g	a	e	a	a	c
64	2022–11–28	22WC105	D	d	d	d	a	d	a	a	d
65	2022–11–28	22WC109	C	c	c	c	a	c	a	a	c
66	2022–11–28	22WC117	D	d	d	d	a	d	a	a	d
68	2022–11–30	22WF252–7P	D	d	d	d	a	d	a	a	d
71	2022–12–02	22WC146	D	d	d	d	a	d	a	a	d
74	2022–12–04	22WC154	C	c	c	c	a	c	a	a	c
79	2022–12–06	22WC167	C	c	c	c	a	c	a	a	c
81	2022–12–07	22WC177	C	c	c	c	a	c	a	a	c
82	2022–12–07	22WC178	D	d	d	d	a	d	a	a	d
83	2022–12–07	22WF296–4P	M	d	c	e	a	c	a	a	c
84	2022–12–09	22WC188	E	a	e	g	a	e	a	a	c
86	2022–12–11	22WC196	C	c	c	c	a	c	a	a	c
87	2022–12–13	22WC200	E	a	e	g	a	e	a	a	c
88	2022–12–13	22WC-208	C	c	c	c	a	c	a	a	c
89	2022–12–14	22WC211	C	c	c	c	a	c	a	a	c
91	2022–12–15	22WC-215	C	c	c	c	a	c	a	a	c
92	2022–12–15	22WF-340–11p	D	d	d	d	a	d	a	a	d
93	2022–12–20	22WC-233	D	d	d	d	a	d	a	a	d
94	2022–12–20	22WC-235	C	c	c	c	a	c	a	a	c

Number	Collection Date	Sample ID	Genotype	PB2	PB1	PA	HA	NP	NA	MP	NS
95	2022–12–22	22WF-378–1p	D	d	d	d	a	d	a	a	d
96	2022–12–23	22WC-244	D	d	d	d	a	d	a	a	d
97	2022–12–23	22WC-250	D	d	d	d	a	d	a	a	d
98	2022–12–22	22WC-254	D	d	d	d	a	d	a	a	d
99	2022–12–25	22WC-247	D	d	d	d	a	d	a	a	d
100	2022–12–25	22WC-252	D	d	d	d	a	d	a	a	d
101	2022–12–26	22WC-245	D	d	d	d	a	d	a	a	d
102	2022–12–26	22WC-265	D	d	d	d	a	d	a	a	d
103	2022–12–27	22WC-281	D	d	d	d	a	d	a	a	d
104	2022–12–27	22WC-278	D	d	d	d	a	d	a	a	d
105	2022–12–28	22WC-284	D	d	d	d	a	d	a	a	d
106	2022–12–28	22WC-280	N	d	c	d	a	d	a	a	d
111	2023–01–03	22WC-317	D	d	d	d	a	d	a	a	d
112	2023–01–02	22WC-326	D	d	d	d	a	d	a	a	d
113	2023–01–02	22WC-328	D	d	d	d	a	d	a	a	d
114	2023–01–02	22WC-329	D	d	d	d	a	d	a	a	d
115	2023–01–02	22WF-429–2p	D	d	d	d	a	d	a	a	d
116	2023–01–03	22WC-324	D	d	d	d	a	d	a	a	d
117	2023–01–04	22WC-345	D	d	d	d	a	d	a	a	d
118	2023–01–05	22WC-336	D	d	d	d	a	d	a	a	d
125	2023–01–07	22WC-357	K	a	c	c	a	c	a	a	c
126	2023–01–06	22WC-363	D	d	d	d	a	d	a	a	d
127	2023–01–09	22WC-365	D	d	d	d	a	d	a	a	d
128	2023–01–09	22WC-366	D	d	d	d	a	d	a	a	d
129	2023–01–10	22WC-372	K	a	c	c	a	c	a	a	c
130	2023–01–09	22WC-373	D	d	d	d	a	d	a	a	d
131	2023–01–10	22WC-376	D	d	d	d	a	d	a	a	d
132	2023–01–10	22WC-378	D	d	d	d	a	d	a	a	d
133	2023–01–11	22WC-374	D	d	d	d	a	d	a	a	d
134	2023–01–12	22WC-390	D	d	d	d	a	d	a	a	d
137	2023–01–13	22WC-401–3P	D	d	d	d	a	d	a	a	d
139	2023–01–13	22WC-394	D	d	d	d	a	d	a	a	d
140	2023–01–11	22WC-405	D	d	d	d	a	d	a	a	d
141	2023–01–13	22WC-406	O	a	c	c	a	c	a	a	a
142	2023–01–16	22WC-413	D	d	d	d	a	d	a	a	d
143	2023–01–16	22WC-412	D	d	d	d	a	d	a	a	d
144	2023–01–17	22WC-421	D	d	d	d	a	d	a	a	d
148	2023–01–20	22WC-426	D	d	d	d	a	d	a	a	d
149	2023–01–20	22WC-427	D	d	d	d	a	d	a	a	d
150	2023–01–22	22WC-428	K	a	c	c	a	c	a	a	c
151	2023–01–23	22WC-432	D	d	d	d	a	d	a	a	d
152	2023–01–27	22WC-453	K	a	c	c	a	c	a	a	c
153	2023–01–06	22WC-464	P	e	d	f	a	c	a	a	b
154	2023–01–30	22WC-460	D	d	d	d	a	d	a	a	d
155	2023–01–30	22WC-468	D	d	d	d	a	d	a	a	d
156	2023–01–30	22WC-471	D	d	d	d	a	d	a	a	d
159	2023–02–03	22WC-491	D	d	d	d	a	d	a	a	d
160	2023–02–05	22WC-492	D	d	d	d	a	d	a	a	d
162	2023–02–06	22WC-502	D	d	d	d	a	d	a	a	d
163	2023–02–07	22WC-500	D	d	d	d	a	d	a	a	d
164	2023–02–13	22WC-522	B	b	b	b	b	b	b	b	b
165	2023–02–15	22WC-529	D	d	d	d	a	d	a	a	d
168	2023–02–28	22WC-572	D	d	d	d	a	d	a	a	d
169	2023–03–03	22WC-586	D	d	d	d	a	d	a	a	d
170	2023–03–04	22WC-590	D	d	d	d	a	d	a	a	d
171	2023–03–07	22WC-597	D	d	d	d	a	d	a	a	d
173	2023–03–07	22WC-599	D	d	d	d	a	d	a	a	d
174	2023–03–06	22WC-603	O	a	c	c	a	c	a	a	a

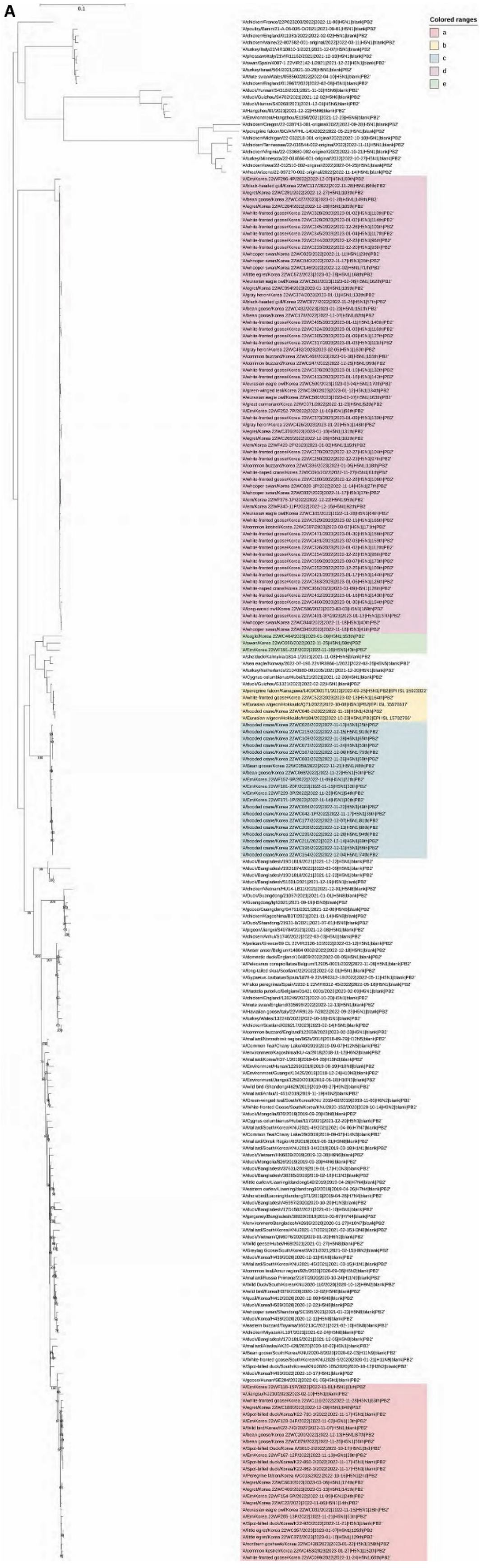
A
0.1
Colored ranges
a
b
c
d
e

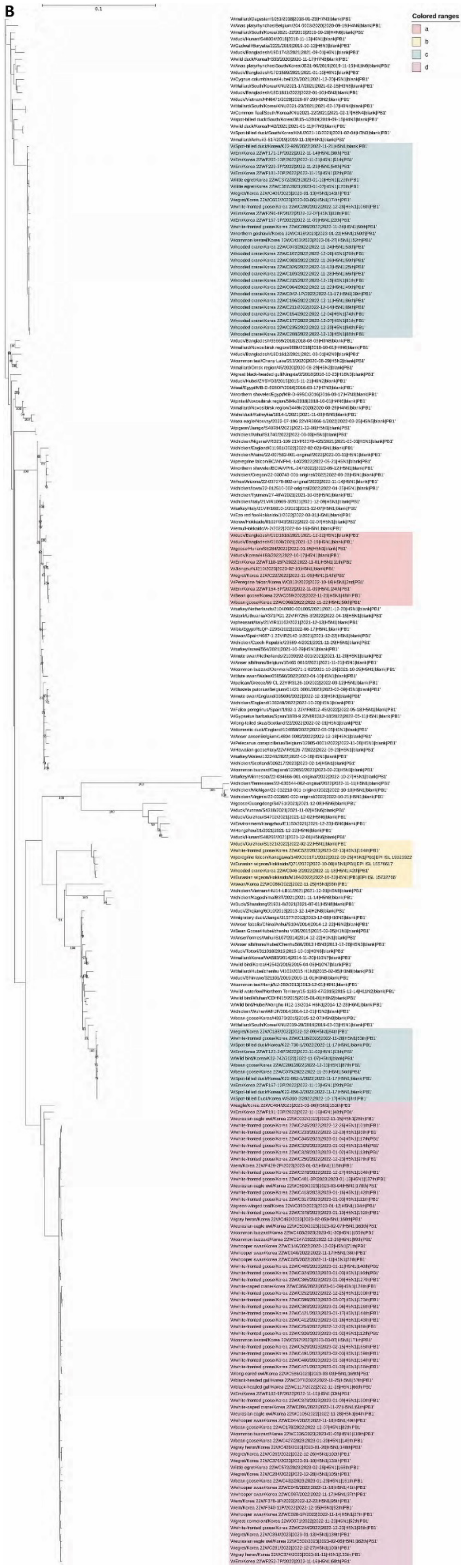
B
0.1
Colored ranges
a
b
c
d

C

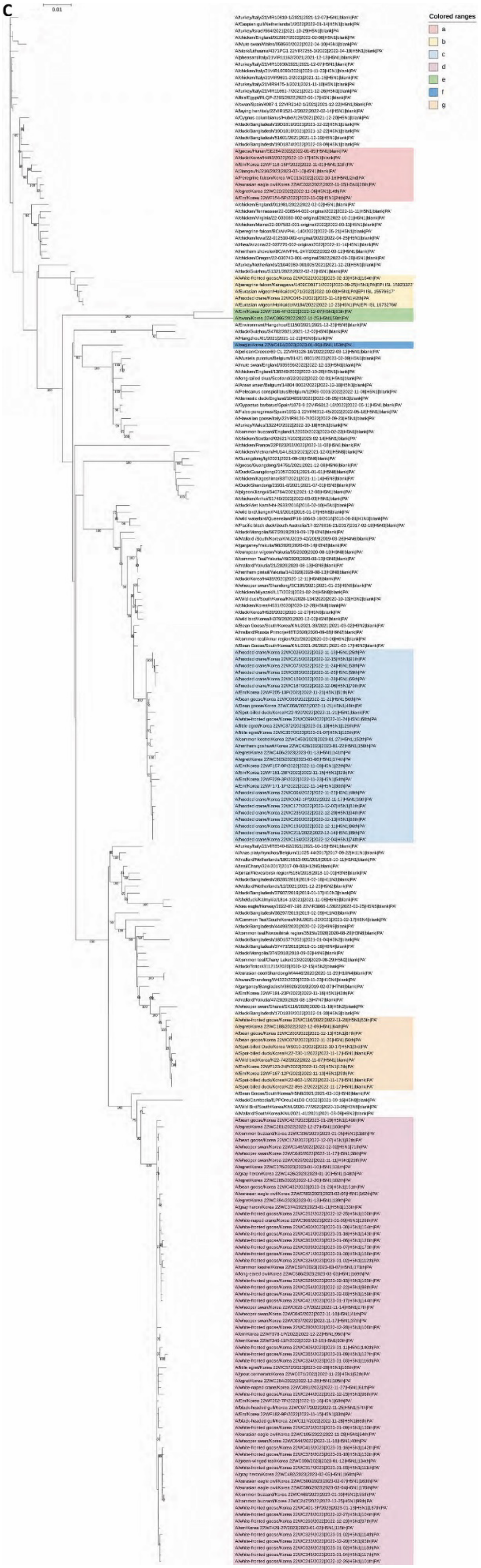

D

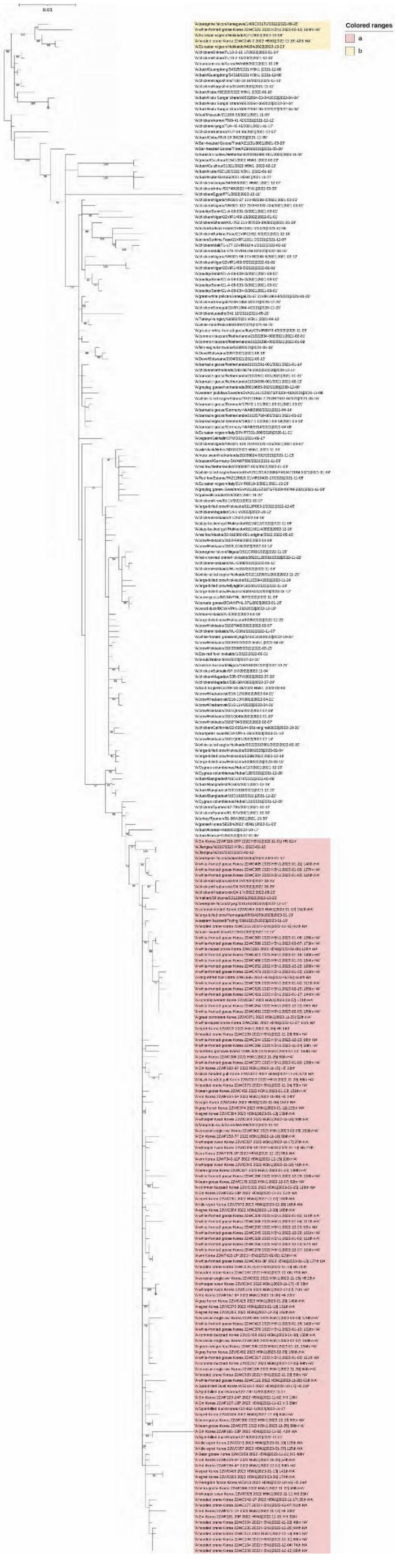

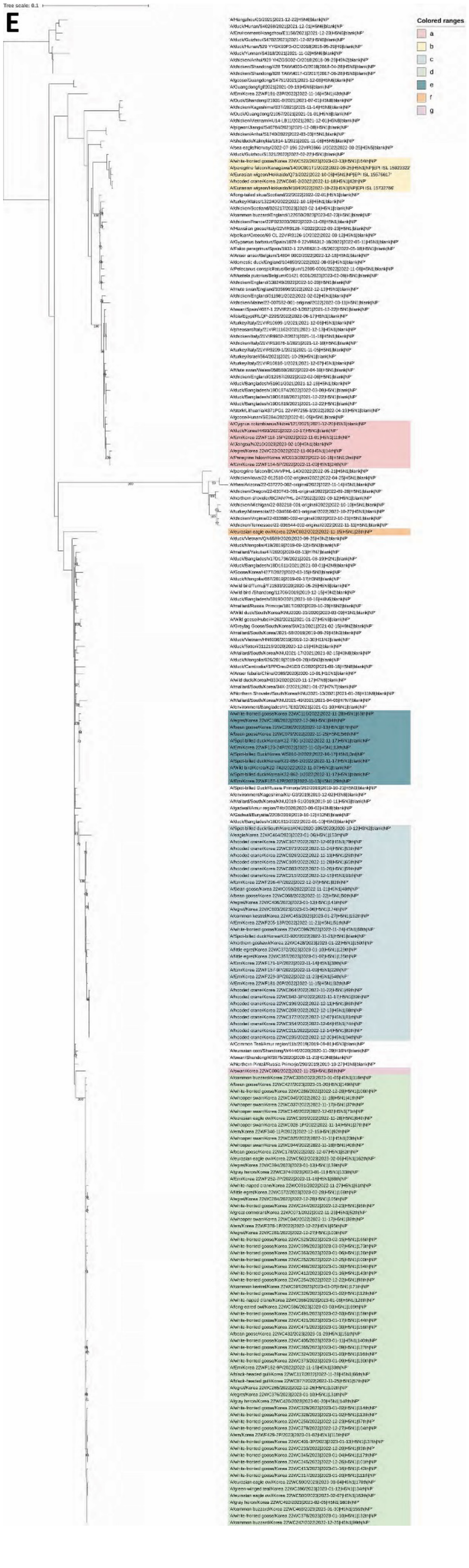
Tree scale: 0.1
E
Colored ranges
a
b
c
d
e
f
g

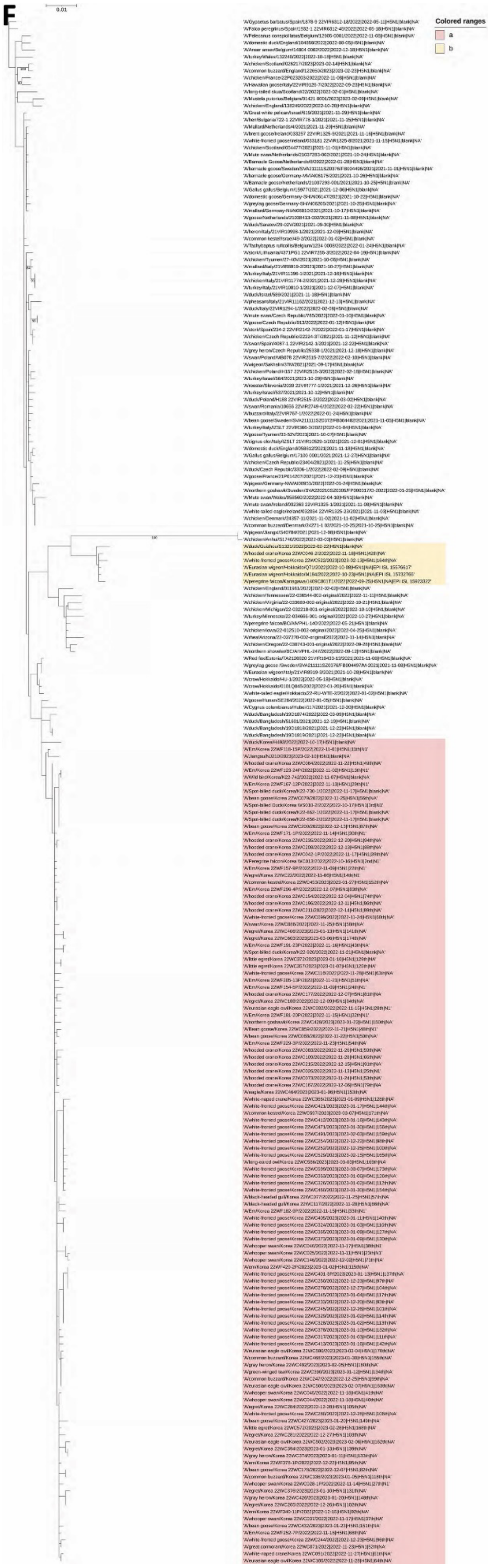
F
0.01
Colored ranges
a
b

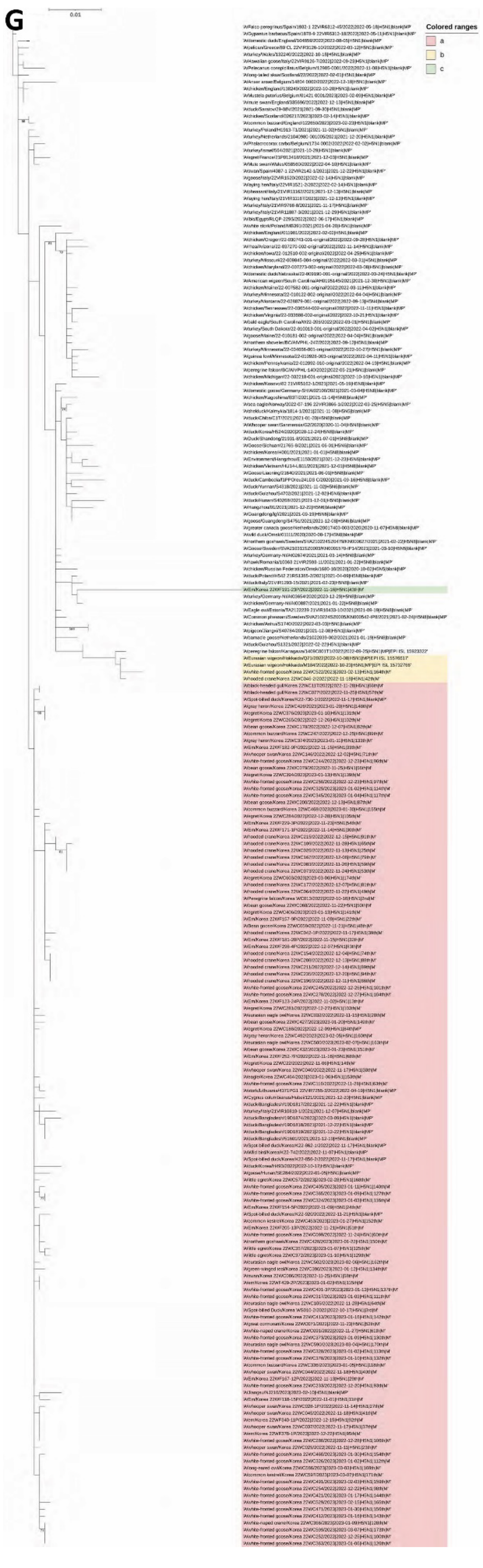
G
0.01
Colored ranges
a
b
c

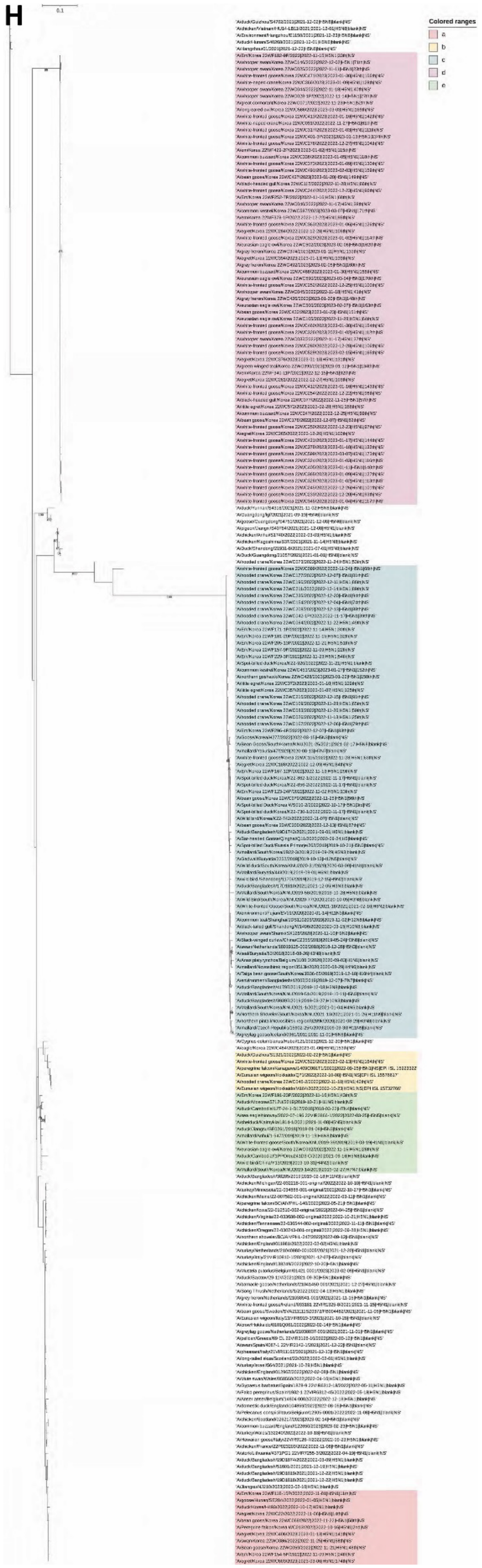

Appendix Figure 1. Maximum-likelihood tree of eight genomes (a) PB2, (b) PB1, (c) PA, (d) HA, (e) NP, (f) NA, (g) MP, and (h) NS of clade 2.3.4.4b H5N1 HPAIV isolated in this study.

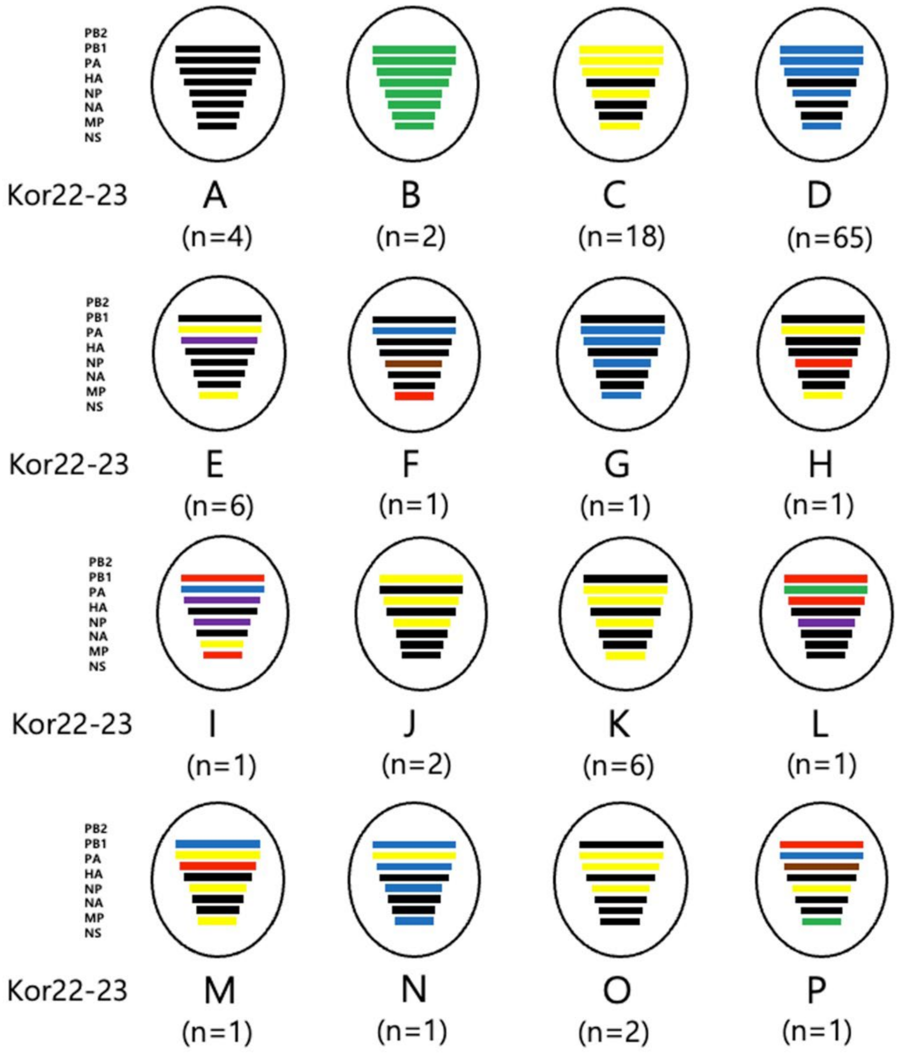

Appendix Figure 2. Schematic of eight viral gene segments of H5N1 clade 2.3.4.4b. Genes are colored according to the phylogenetic tree analyses and classification.

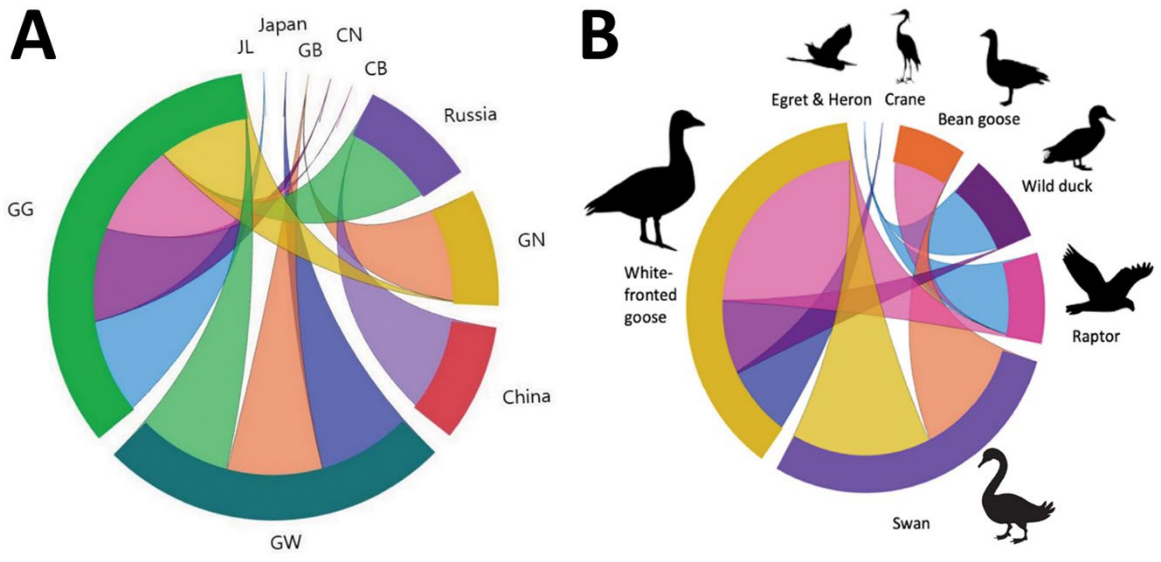

Appendix Figure 3. Chord diagram representing the transmission network of (a) geographic location and (b) host type. The length of the arcs represents the migration rate of the originating trait.
